# Toxicological problems of tattoo removal: characterization of femtosecond laser-induced fragments of Pigment Green 7 and Green Concentrate tattoo ink

**DOI:** 10.1007/s00204-024-03953-6

**Published:** 2025-01-15

**Authors:** Elvira Maria Bauer, Cosimo Ricci, Daniele Cecchetti, Giorgia Ciufolini, Daniel Oscar Cicero, Marco Rossi, Ettore Guerriero, Stefano Orlando, Marilena Carbone

**Affiliations:** 1 Institute of Structure of Matter, Italian National Research Council (ISM-CNR), c/o Area della Ricerca di Roma1, Strada Provinciale 35d n. 9, Montelibretti, 00010 Rome, Italy; 2 STARTNETICS – Department of Chemical Science and Technologies, University of Rome Tor Vergata, Via della Ricerca Scientifica 1, 00133 Rome, Italy; 3 Department of Basic and Applied Sciences for Engineering (SBAI), Sapienza University of Rome, Via Antonio Scarpa 16, 00161 Rome, Italy; 4 Research Center on Nanotechnologies Applied to Engineering (CNIS), Sapienza University of Rome, P.le Aldo Moro 5, 00185 Rome, Italy; 5 Institute for Atmospheric Pollution Research, Italian National Research Council (CNR-IIA), c/o Area della Ricerca di Roma1, Strada Provinciale 35d n. 9, Montelibretti, 00010 Rome, Italy; 6 Institute of Structure of Matter, Italian National Research Council (ISM-CNR), FemtoLAB, C.da S. Loja, 85050 Tito Scalo, Italy

**Keywords:** Femtosecond laser treatment, Pigment PG7, Green tattoo ink, Fragmented moieties hazard, NLO mechanisms, Fibers generation

## Abstract

Femtosecond lasers represent a novel tool for tattoo removal as sources that can be operated at high power, potentially leading to different removal pathways and products. Consequently, the potential toxicity of its application also needs to be evaluated. In this framework, we present a comparative study of Ti:Sapphire femtosecond laser irradiation, as a function of laser power and exposure time, on water dispersions of Pigment Green 7 (PG7) and the green tattoo ink Green Concentrate (GC), which contains PG7 as its coloring agent. The treated samples were subsequently analyzed via UV‒Vis spectroscopy, gas chromatography‒mass spectrometry (GC‒MS), SEM imaging and associated statistical analysis. We found that, on average, the discoloration efficacy of femtosecond laser treatment was comparable to that of nanosecond lasers as were the decomposition products. In fact, two primary types of fragments are produced, both of which are potentially harmful, resulting either from the decomposition of chlorinated phthalocyanine (i.e., PG7) or from the active chlorination of naphthalene impurities. However, the outcomes for the PG7 and GC treatments differed significantly from each other from several points of view. The spectral intensity patterns of GC and PG7 were distinct, depending on the treatment conditions, and showed linearity with power only in the case of GC. Additionally, the relative ratios of the fragment products differed significantly, with the production rate showing a linear dependence on power only in the case of GC and no discernible trend for PG7. Shape and size distribution of the generated particles were highly dependent on the type of sample. Femtosecond laser irradiation of GCs primarily produces nanoparticles with a homogeneous size distribution, which are typically considered nontoxic. Large aggregates also formed, exhibiting a regular shape. In contrast, PG7 yielded rods and needles with aspect ratios similar to those of toxic fibers.

**Supplementary Information:**

The online version contains supplementary material available at 10.1007/s00204-024-03953-6.

## Introduction

With nearly 50% of the population in the United States, Italy, and Sweden bearing at least one tattoo, the demand for its removal has risen by 32% since 2011 (Egozi and Toledano 2024). The typical procedure involves laser treatments involving the use of Q-switched devices operating at different wavelengths and pulse durations. Their application has been investigated in several configurations and contests, depending on color for removal, with the aim of achieving the highest decolorization efficacy. Green, of all colors, has always been considered the most difficult to treat since it requires several sessions of treatment for complete removal via Nd:YAG nanosecond lasers (Adatto 2004). However, the availability of Nd:YAG picosecond lasers on the market has changed this scenario since they display largely increased efficacy in the treatment of green inks as evidenced by many investigations (Wu et al. 2021; Gurnani et al. 2022; Cecchetti et al. 2022a; Baleisis and Rudys 2023). Given the improvements in removal effectiveness, when moving on from nano- to picosecond lasers, the inevitable question is whether lasers with even shorter pulses would provide even more efficient removal. In this framework, femtosecond lasers have attracted increasing attention because of their high peak power, which might induce strong linear as well as nonlinear optical effects on the chromophore of the ink, causing fast molecular rearrangements of the pigment and resulting in efficient fragmentation pathways and consequent prompter discoloration. Moreover, it is important to determine whether such treatments induce the same type of fragmentation and/or reaction as the other lasers do or whether they can be associated with a lower toxicity of the final products. The potential efficacy of femtosecond lasers, however, needs to be offset against their major drawback, i.e.*,* the typical operational wavelength of 800 nm (*e.g.,* Ti:Sapphire), which falls on the tail of the absorption of the pigments, thus diminishing the initial boost of the fragmentation processes. In this context, comparative studies using lasers with different pulse durations are desirable since they help provide insights into both the fundamental processes involved and their application in tattoo removal. To date, the effects of irradiation with nano-, pico-, and femtosecond lasers have been investigated by Belikov et al. on two substrates: a cotton fabric colored with black tattoo ink and a pig skin extract after deposition of the same black ink (Belikov et al. 2019). The discoloration was observed upon treatment with all three lasers. Furthermore, the fluence corresponding to the ablation threshold decreases with increasing laser pulse, with values of Q = 3.3, 3.0, and 0.8 J cm^–2^ for the nano-, pico-, and femtosecond pulses, respectively, thus indicating the greater efficacy of the latter.

As far as green inks are concerned, comparative studies were previously performed by our research group, using Ruby nanosecond and Nd:YAG nanosecond and picosecond lasers on aqueous dispersions on the ink Green Concentrate by Eternal (Cecchetti et al. 2022a). Furthermore, the production of toxic fragments from the laser and thermal treatments of phthalocyanine-based pigments was thoroughly investigated both for molecular breakage and for nanoparticle aggregation and downsizing.

In the present study, we performed a thorough investigation on the removal efficiency of a Ti:sapphire femtosecond laser on aqueous dispersions of the same ink, Green Concentrate (labeled as GC), as well as the pigment that it contains, i.e., PG7, a hexadecachloro copper phthalocyanine. We opted for a comparative analysis of pigment and ink to determine whether significant differences can be found in the decomposition patterns when these patterns are treated with a femtosecond laser. In particular, we investigated whether phthalocyanine decomposition would occur completely into gaseous moieties due to the high power density, triggering new decomposition mechanisms, or whether toxic chlorinated fragments would still be generated upon treatment. The morphology of the generated particles is also an issue because of the possible formation of rods and fibers, with aspect ratios associated with high toxicity (Schröder et al. 2014). To assess various aspects related to the femtosecond treatments, investigations were carried out at different peak powers with fixed durations, as well as at varying durations with a fixed peak power. The analyses were carried out via UV‒Vis spectroscopy, gas chromatography‒mass spectrometry, and associated principal component analysis (PCA) of the fragments, as well as SEM imaging.

## Materials and methods

The green tattoo ink (Green Concentrate) examined in this work is based on the polychlorinated copper phthalocyanine pigment PG7. Aqueous dispersions of both the PG7 pigment (Kremer GmbH) and the Green Concentrate tattoo ink (Eternal Ink Ltd.) at a concentration of 0.09 mg/mL were prepared. PG7 pigment (15.6 mg) was stirred in 173 mL ultrapure H_2_O (HPLC grade, Carlo Erba) for several days and subsequently sonicated in an ultrasonic bath (Branson) until a homogeneous green dispersion was obtained. In addition, 15.3 mg of air-dried green ink concentrate was dispersed in 170 mL of ultrapure H_2_O by magnetic stirring for 1 h. Five milliliters of each dispersion was then transferred with glass pipettes in thoroughly cleaned glass vials. A blank sample of 5 ml ultrapure water was prepared in the same manner. Contact with plastic materials was avoided to avoid side contamination.

### Laser setup and irradiation parameters

A Spectra Physics Ti:sapphire laser (max. average power 3 W) capable of generating 100 fs laser pulses with a maximum pulse energy of 3 mJ at 800 nm was used to treat the water dispersions of PG7 and GC inks (0.09 mg/mL). An irradiation wavelength of 800 nm was chosen because this represents the highest available pulse energy. The experimental laser setup also allows wavelength tuning from 290 to 2500 nm but at a lower pulse energy. A custom vial cap with a quartz window was used to allow the laser to pass through a flat quartz surface inside the closed vials. The laser was focused in the center of the vial, 1 cm below the surface of the solution.

Two different irradiation experiments were set up. All three samples (PG7, GC ink and plain water) were treated for 25 min, and the power of the laser pulse was varied from 3 to 0.5 W to establish the ideal experimental parameters for future treatments. Afterward, the samples were irradiated at the highest power, i.e., 3 W, at different irradiation times, reaching 150 min in the case of the PG7 dispersion. Coloration, fragmentation, and morphology of the exposed samples were evaluated via UV‒Vis, GC‒MS, and SEM techniques.

The experimental setups of the laser treatments for the different samples are summarized in Table 1. The names of the treated samples are assigned a capital letter followed by two numbers: the letter G indicates the green concentrate ink, the letter P indicates the pigment PG7, and the letter W indicates the water; the first number indicates the duration of the treatments in minutes, and the second number is the power used in the treatment expressed in W.

**Table 1:** Radiation intensity and exposure times used for the treatments of GC, PG7, and reference water by the femtosecond laser

Time/min	Intensity/W	GC	PG7	Water
5	3	G5-3	P5-3	W5-3
10	3	G10-3	P10-3	W10-3
20	3	G20-3	P20-3	W20-3
25	0.5	G25-0.5	P25-0.5	
25	1.25	G25-1.25	P25-1.25	W25-1.25
25	2.25	G25-2.25	P25-2.25	W25-2.25
25	3	G25-3	P25-3	W25-3
30	3	G30-3	P30-3	W30-3
60	2.25	–		
150	2.25	–	P150-2.25	

### UV‒Vis spectra

Immediately after the laser treatments, the supernatants of the samples were transferred into 10 mm optical path length quartz cuvettes, and the UV‒Vis spectra were recorded with a PerkinElmer Lambda 950 UV/VIS/NIR spectrophotometer. UV‒vis spectra of the deep green GC dispersions treated for 20 min with 3 W femtosecond laser pulses corresponding to a total pulse energy of 3.6 kJ were also examined with an optical path length of 1 mm.

### Gas chromatography‒mass spectrometry (GC‒MS): extraction, analysis, and identification

Prior to GC‒MS, extraction of the aqueous samples in ethyl acetate was performed. Specifically, 1 mL of each femto-treated sample was placed in a clean 5 mL glass vial, 1 mL of ethyl acetate was added, and the mixtures were stirred for 18 h at 640 rpm with a magnetic stirrer (IKA ® KS 130). Then, 0.7 mL of the ethyl acetate extract was removed, and the mixture was stored in a refrigerator. The extraction process was repeated three times. Anhydrous sodium sulfate (Carlo Erba Reagents) was added to the unified ethyl acetate extracts, and 1 mL of each anhydrous extract was stored in the refrigerator before GC‒MS/MS analysis. Each extract was further concentrated to 10 μl by a gentle flow of nitrogen gas and spiked with completely deuterated anthracene (D10) and perylene (D12) as internal standards at a concentration of 1 ng/μL. The GC‒MS analysis was carried out with a Trace GC Ultra gas chromatograph (Thermo Scientific, Waltham, MA, USA) equipped with a TriPlus autosampler unit and coupled to a TSQ Quantum Triple Quadrupole MS‒MS/MS spectrometer (Thermo Scientific, Waltham, MA, USA). The column was an XLB-ms fused silica capillary column (Varian, Inc.), 60 m × 0.25 mm, i.d. 0.25 μm film thickness. Hydrogen was employed as the carrier gas at a 3 mL min^−1^ flow rate. One microliter of solution was injected in splitless mode at 250 °C. The oven program consisted of an isotherm at 90 °C for 5 min and a temperature ramp of 10 °C min^−1^ up to 280 °C, which was held for 5 min. The MS was operated in positive electron ionization (EI +) mode, with an electron energy of 70 eV and an emission current of 50 μA. The acquisition was in scan mode in the range of 35–600 m/z in 0.2 s. The transfer line and the ion source temperatures were maintained at 290 °C and 300 °C, respectively.

The data from GC‒MS were compared with the NIST and Schreiver databases (Schreiver et al. 2019) to identify the chemical nature of every fragment detected in the chromatograms. An identification analysis was conducted on the compounds detected in all the chromatograms of the laser-treated PG7 and GC samples, the results of which are listed in Table 2. A semiquantitative analysis was then conducted on the selected fragments. For all molecules, the integrated peak areas from the chromatogram, corresponding to the mass of the primary fragment, were normalized against the peak area of the anthracene-d10 standard. The normalized integrals were also used, with unit-variance scaling, for the principal component analysis (PCA) performed with Python v3.9.12 via the Sklearn v1.0.2 library.

**Table 2:** List of fragments present in all samples upon femtosecond laser treatment of GC ink and PG7, along with associated acronyms

Common fragments	Acronym
Pentachlorobenzene	PVB
Trichlorobenzonitrile	TCBN*
Tetrachlorobenzonitrile	TeCBN
Pentachlorobenzonitrile	PCBN
Tetrachlorobenzodinitrile	TeCBDN
Tetrachlorobenzaldehyde	TeCBA
1,4-Dichloronaphthalene	1-4DCN
2,7-Dichloronaphthalene	2-7DCN
2,3,6-Trichloronaphthalene	2–3-6TCN
1,3,7-Trichloronaphthalene	1–3-7TCN
Tetrachlorophthalimide	TeCF
4,5,6,7-Tetrachloro 2,3-dihydro-1H-isoindole	TeCHI

*Detected only in GC ink

### SEM imaging

The morphology of the samples after treatment was determined by depositing a drop on a silicon wafer used as a sample holder and imaging it via field emission scanning electron microscopy (FE-SEM) (Field Emission Scanning Electron Microscope SUPRA TM 35, Carl Zeiss SMT, Oberkochen (Germany)) operating at voltages between 1.5 and 7 kV. Statistical analysis was performed with ImageJ software after the areas to be examined were selected and random manual counting of a few areas for control purposes was performed.

## Results

The difficulty of fully removing green tattoo pigments remains a central challenge in tattoo treatments, along with the potential harm of generated fragments and particles. Although advances from nanosecond to picosecond laser treatments have shown promising improvements, complete removal often still requires multiple sessions. Moving on to femtosecond lasers, which could enable more efficient pigment breakdown, could result in faster and more effective decolorization. However, simultaneously, the production of harmful molecules or the aggregation of particles with aspect ratios close to dangerous ones needs to be monitored. This study was conducted in two phases. First, the discoloration efficiency was evaluated to determine how it compares to currently adopted treatments and, as a consequence, whether higher or lower demanding conditions are necessary. Afterward, the production of potentially harmful fragments was monitored as were the morphology of the produced particles, their aspect ratio, and their abundance. The investigation has been carried out both on green concentrate ink and on the pigment that it contains, i.e.*,* PG7. Furthermore, investigations were carried out at different peak powers with fixed durations as well as at varying durations with a fixed peak power. Comparative studies were also performed on the basis of prior research in our laboratory with other laser types. The analyses are carried out via UV‒Vis spectroscopy, gas‒chromatography mass spectrometry, and associated PCA of the fragments, as well as SEM imaging.

### UV‒Vis spectra

**Fig. 1:**
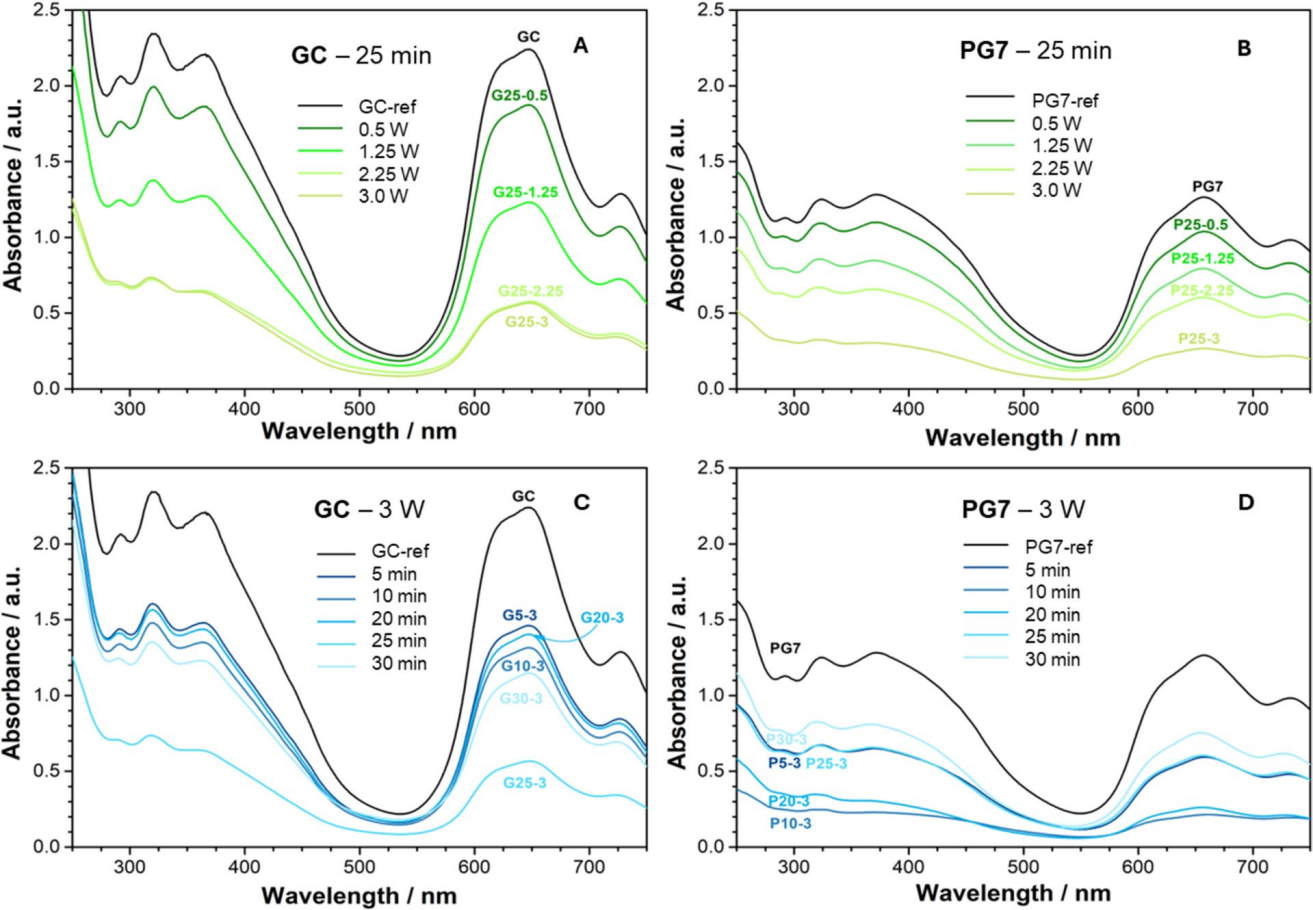
UV‒Vis spectra of **A** the GC dispersions irradiated for 25 min at different powers, and **B** the PG7 dispersion irradiated for 25 min at different powers, **C** the GC dispersions treated with 3 W of power for various durations and **D** the PG7 dispersion irradiated with 3 W of power for various durations

UV‒Vis spectra were taken for all treated samples and for the reference untreated GC and PG7 samples as shown in the four panels of Fig. 1. The spectra are grouped by increasing the laser power at a constant exposure time (Fig. 1A, B) and by increasing the exposure time at a constant power (Fig. 1C, D). The spectra of the GC ink and PG7 before treatment display the typical Soret and Q bands of chlorinated copper phthalocyanines in the ranges of 300–450 nm and 550–750 nm, respectively. The overall intensity of the bands varies with laser irradiation, but the phthalocyanine features, i.e., the B and Q bands, are largely preserved. This implies that the treatments either provoked the fragmentation of the macrocycle into smaller moieties that do not absorb in the UV‒Vis region or affected the aggregation state of the phthalocyanine agglomerates but did not induce partial degradation, resulting in an alteration in the Q/B band ratio.

From these analyses, two distinguishing features characterize the UV‒Vis spectra of both reference and femtosecond-treated samples. Notably, the spectrum intensity of PG7 is lower than that of the GC ink despite PG7 being a pure pigment, whereas the GC ink contains PG7 diluted with additives and binders. This discrepancy can be attributed to two factors: commercial PG7 although considered pure, may contain additives at unknown concentrations that could exceed those found in the ink formulation. Additionally, the greater hydrophobicity of PG7 than that of the GC ink makes its dispersion more susceptible to particle deposition, effectively diminishing its concentration in the solution.

Furthermore, a second important feature is that the spectral intensity varies linearly with the irradiation power when the duration is held constant at 25 min. In contrast, when the power is fixed at 3 W, the intensity tends to fluctuate as the exposure time increases.

The decrease in the spectral intensity as a function of irradiation power is straightforward since it can be correlated with macrocycle fragmentation into smaller moieties. From a purely mechanistic point of view, this type of correlation suggests a nonlinear optical (NLO) response (as also indicated by the pinkish color around the focus in Fig. SI1 of the Supplementary Information) under the condition of Saturable Absorption (SA) (Li and Li 2012).

To evaluate the discoloration efficiency and compare the Ti:sapphire femtosecond laser with the Nd:YAG nano-/pico- and Ruby nanolasers, samples of GC ink were treated with each of the three laser types.

**Fig. 2:**
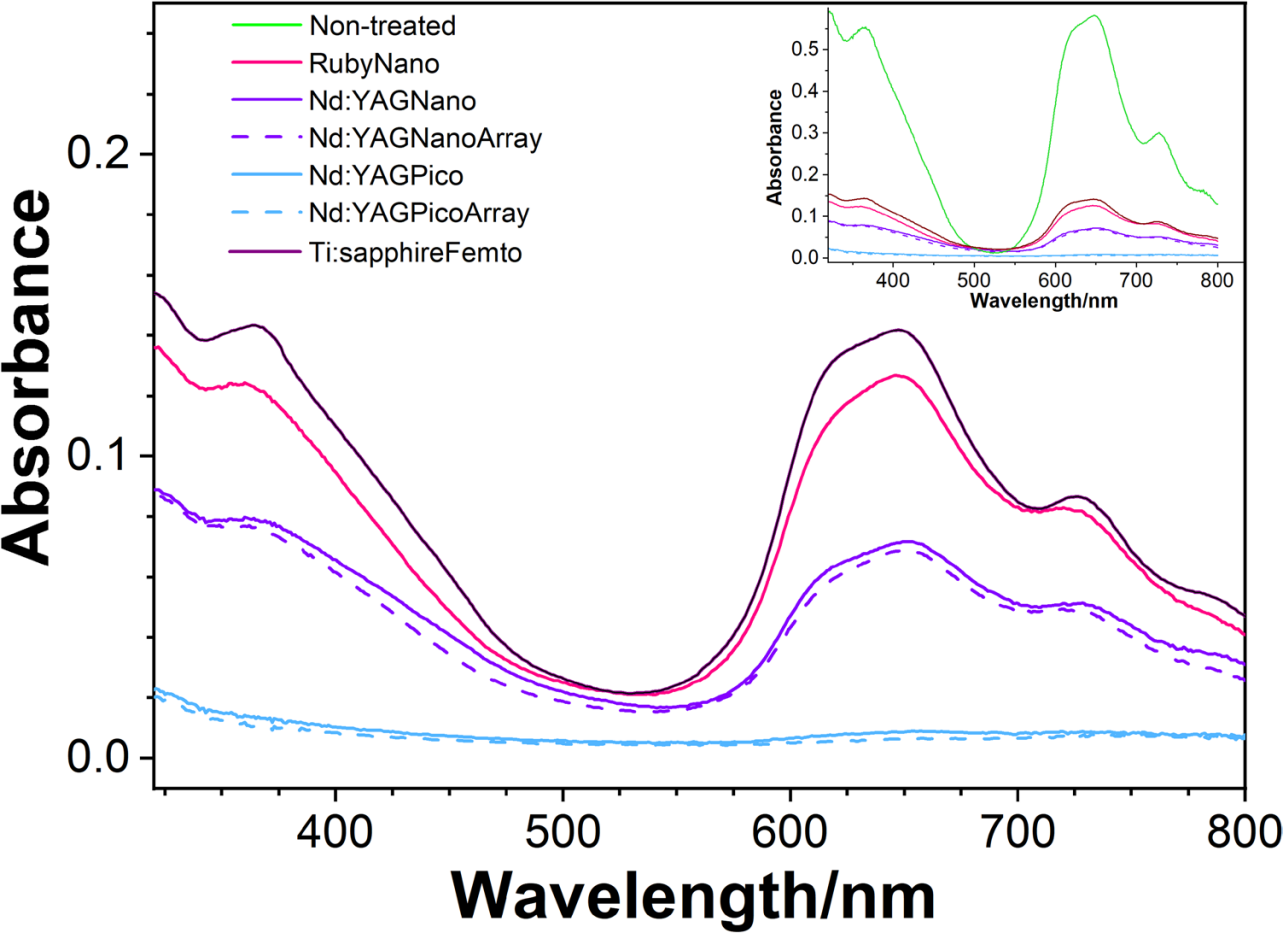
UV–Vis spectra of the GC ink dispersion upon laser treatment: dark violet solid line = Ti:Sapphire femtosecond laser, red solid line = nanosecond ruby laser, light violet solid line = nanosecond Nd:YAG, light violet dashed line = nanosecond Nd:YAG with array, light blue solid line = picosecond Nd:YAG, light blue dashed line = picosecond Nd:YAG with array. In the inset, the same set of spectra is plotted along with the nontreated sample, reported with a green solid line

Figure 2 shows the absorption spectra of samples treated with the Ti:sapphire femtosecond laser (800 nm) and compares them to the spectra of the samples treated with the Nd:YAG nano-/pico- (532 nm) and Ruby (694 nm) nanolasers obtained from previous experiments (Cecchetti et al. 2022c). The experiments were all conducted under the same conditions, using samples of the same ink and concentration, and subjected to treatments with the same total irradiation energy, after control of the composition as performed by Bauer et al. (Bauer et al. 2019).

These comparisons indicate that the intensity of the spectrum of the GC ink treated with the femtosecond laser is comparable to that of the sample treated with the ruby laser despite high peak power and narrow time structure of the femtosecond laser pulse. To consider the discoloration of GC dispersions treated with a femtosecond laser compared with those treated with Ruby nano-, Nd:YAG nano-, and picosecond lasers, some considerations regarding the decomposition mechanism of phthalocyanine and the effective volume irradiated are necessary.

Since the spot sizes of the laser beam and the vertical dispersion (albeit Gaussian) are different for the three types of lasers, being 2 cm for the Nd:YAG (both nano and pico) and Ruby beams and 1 cm for the Ti:Sapphire beam, the total volume of the GC of interest for the primary beam is also different, with a V_N_:V_R_:V_Ti_ ratio of 37.5 mm^3^:37.5 mm^3^:10.5 mm^3^, where V_N_, V_R_, and V_Ti_ are the volumes affected by the Nd:YAG, Ruby and Ti:Sapphire beams, respectively (in conic approximation). Therefore, even when all phthalocyanine molecules in the focus of the Ti:sapphire beam are decomposed, their total amount remains 3.5 lower than that of the Ruby laser.

Additional considerations can be made regarding the overall mechanisms of decomposition. Typically, when immersed in a solvent, chromophores such as phthalocyanines trigger two additional types of phenomena leading to decomposition: cavitation and photothermally induced bond breakage. Although present in all cases, the extent and the efficacy of the phenomena are determined by the wavelength of the laser beam and the absorption spectrum. The lasing of Nd:YAG is carried out at 532 nm, which corresponds to an absorption minimum of the phthalocyanine spectrum as it is the region at 800 nm, where Ti:sapphire lases. The latter, however, is in the infrared region, whose absorption excites vibrational states, thus largely contributing to photothermal decomposition. Notably, additional mechanisms of fast and efficient photothermal conversion are active in solids such as nanoparticles through exciton–exciton annihilation, which results in a high density of excited molecules (Hosokawa et al. 2000). The wavelength of the ruby laser at 694 nm, instead, is the closest to the maximum of the Q band at 646 nm and is more likely to trigger direct decomposition upon electronic excitation.

### GS‒MS fragment identification

GC‒MS analysis revealed twelve fragments common to nearly all samples following femtosecond laser treatment with GC ink and PG7, all of which were identified as harmful (Bauer et al. 2022, 2021, 2020; Cecchetti et al. 2022), as illustrated in Table 2, along with their respective acronyms. Trichlorobenzonitrile (TCBN) was not detected in the PG7 samples.

Overall, these compounds can be divided into two different groups: those related to the decomposition of chlorinated phthalocyanine and those stemming from the active chlorination of naphthalene impurities.

The first group comprises chlorinated benzodinitriles, benzonitriles, phthalimides, benzaldehydes, or benzenes. However, some of the fragments were already present in the native GC ink before laser treatment. In particular, pentachlorobenzene and tetrachlorophthalimide were detected in the H_2_O/ethyl acetate and acetone extracts, whereas pentachlorobenzonitrile was detected in the chloroform extract (Bauer et al. 2022).

The second category is represented by chlorinated naphthalenes, such as 1,4-dichloronaphthalene, 2,7-dichloronaphthalene, 2,3,6-trichloronaphthalene, and 1,3,7-trichloronaphthalene. These compounds likely do not result from phthalocyanine decomposition. Instead, they appear to form through chlorination during laser treatment of preexisting naphthalene or methylnaphthalene impurities present in the original ink (Bauer et al. 2022). This hypothesis is supported by the common use of naphthalenesulfonic acid and its derivatives as surfactants or grinding additives in pigment preparations (Herbst and Hunger 2004).

Interestingly, the types of fragments detected upon femtosecond laser treatment are quite similar to those detected after irradiation with Nd:YAG nanosecond and picosecond lasers (532 nm) at the same total irradiation energy, especially chlorinated naphthalene, tetrachlorobenzodinitrile, dichloro- or trichlorobenzonitriles and tetrachloroisoindolines (Cecchetti et al. 2022). This correspondence strongly supports similarities in the fragmentation mechanism, independent of the excitation wavelength or pulse duration. Instead, chlorinated benzaldehyde is detected solely upon femtosecond laser treatment; however, it might be a derivative of 4-methylbenzaldehyde, which is also present in the native ink composition, rather than a product of phthalocyanine decomposition.

### GC‒MS fragment intensity trends with time and power

**Fig. 3:**
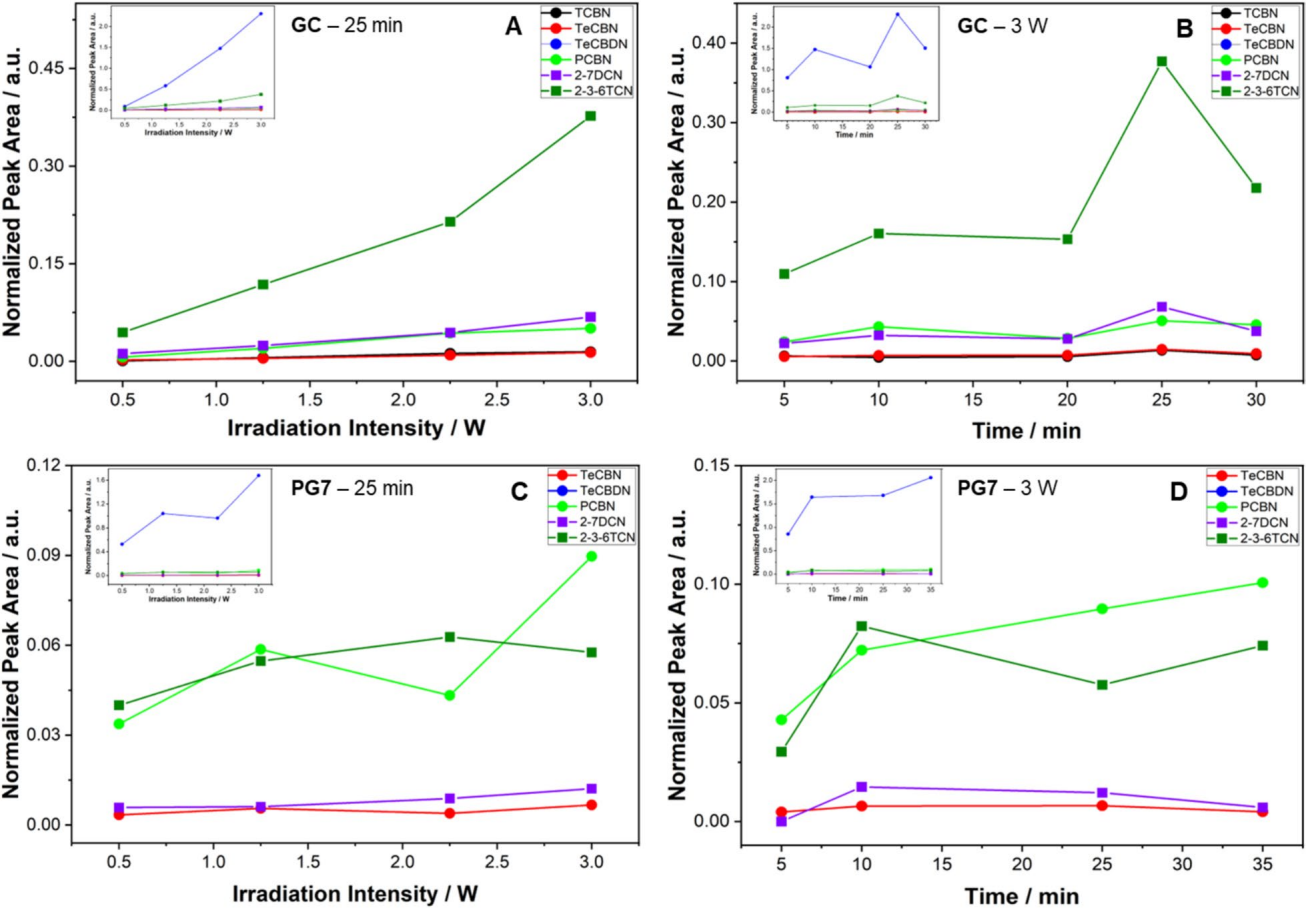
Chromatographic peak area of selected fragments normalized to the internal standard, as a function of laser power at a fixed irradiation time (25 min), in **A** and **C** for the GC ink and PG7 pigment, respectively, and as a function of irradiation time at a fixed laser power (3 W), in **B** and **D** for the GC ink and PG7 pigment, respectively. The dots indicate fragments from the decomposition of chlorinated phthalocyanine, and the squares indicate those from the active chlorination of naphthalene impurities

A semiquantitative analysis was performed by plotting the normalized peak areas of different fragments as a function of irradiation time at a fixed laser power (3 W) and as a function of laser power at a fixed irradiation time (25 min). These plots were obtained for GC ink, focusing on six selected fragments: trichlorobenzonitrile (TCBN), tetrachlorobenzonitrile (TeCBN), tetrachlorobenzodinitrile (TeCBDN), pentachlorobenzonitrile (PCBN), 2,7-dichloronaphthalene (2,7-DCN), and 2,3,6-trichloronaphthalene (2,3,6-TCN). For the PG7 pigment, five of these fragments were identified, with TCBN not being detected. Figure 3 illustrates the trends for the selected fragments, highlighting their behavior under the specified experimental conditions. To enhance clarity, the TeCBDN fragment, which showed higher intensity in all the graphs, is presented only in the inset. This approach allows the main plots to emphasize the remaining fragments without signal overlap, using a customized scale for each graph to achieve this separation.

The analysis of the GC samples exposed for 25 min to different power levels revealed a linear trend in the intensity of the fragmentation products as the applied power increased (Fig. 3A) for both classes of fragments. The observed linear behavior is supported by high values of the R^2^ coefficient obtained for each of the six fragments, indicating a strong correlation between the applied power and the production rate of the fragments. Specifically, the R^2^ values for the fragments are as follows: trichlorobenzonitrile (0.987), tetrachlorobenzonitrile (0.9814), tetrachlorobenzodinitrile (0.9902), pentachlorobenzonitrile (0.9824), 2,7-dichloronaphthalene (0.979), and 2,3,6-trichloronaphthalene (0.9588). Conversely, when the GC ink samples were treated at a constant power of 3 W for increasingly longer exposure times, no correlation was observed with the intensity trend of these fragments (Fig. 3B).

In contrast to the GC ink, which exhibited a strong linear correlation between all fragments and the applied laser power, the PG7 pigment showed no such dependence for most fragments (Fig. 3C). The lower R^2^ values for the PG7 pigment are likely attributable to its instability in dispersion. Notably, only the TeCBDN and 2,7-DCN fragments are correlated with the applied power, with R^2^ values of 0.8012 and 0.9114, respectively. Under constant power conditions, the time-dependent analysis of PG7 revealed that only the TeCBDN and PCBN fragments linearly increased over time, with corresponding R^2^ values of 0.7257 and 0.8769, respectively (Fig. 3D).

The analysis of the fragments indicates that TeCBDN is commonly identified in both pigment and ink samples following laser treatments, suggesting a direct association with the decomposition of the PG7 pigment. Furthermore, there is a noticeable dependence on the applied laser power for the production of this fragment in both samples.

4,5,6,7-Tetrachloro-2,3-dihydro-1H-isoindole is detected in all PG7 samples, whereas in the GC ink samples, it is only observed following treatment with high power or after prolonged exposure times, suggesting that its formation is occasionally below the limit of detection (LOD) of the GC‒MS method.

**Fig. 4:**
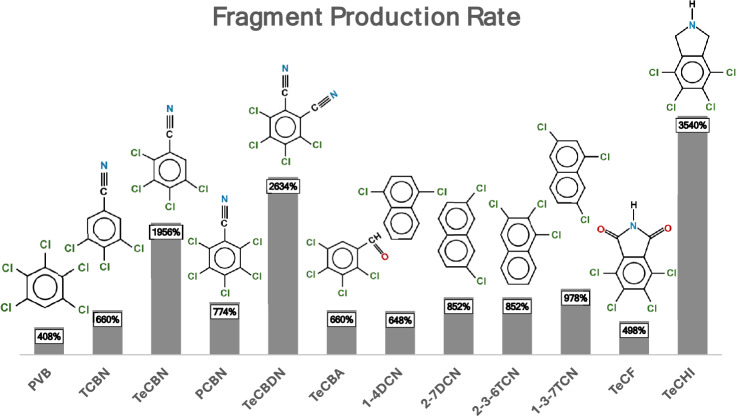
Production rates of all the fragments, calculated for the GC ink samples treated for 25 min at 3 W and 0.5 W, along with their respective chemical structures. The 100% threshold indicates equal production rates at 3 W and 0.5 W

Given the observed linear increase in the production of all fragments with applied power in the GC ink analysis, the magnitude of this increase was evaluated for each of them. This was accomplished by calculating the production rate of all fragments via the normalized peak area at 3 W divided by that at 0.5 W. The results of this analysis are presented in Fig. 4.

The production rate of all common fragments was calculated according to formula (1):

1 $$PR=\left(\frac{P{A}_{3W, 25 min}}{{PA}_{0.5W, 25 min}}\right)\%$$

where PR is the production rate and PA is the peak area for the 25-min treatments at 3 W and 0.5 W, respectively. These values are indicative of the dependency of fragment production on the pulse power in W.

The production rate quantifies the extent to which the intensity of a specific fragment’s production increases with applied power. A PR of 100% indicates that the fragment production rate at 3 W matches that at 0.5 W. Higher values indicate a percentage increase in the production rate under higher power conditions relative to 0.5 W treatment, which is taken as the reference.

Although all fragments exhibit some degree of increase, the TeCHI, TCBN, and TeCBDN fragments show the most pronounced increase in production as the applied power increases. These fragments maintain the integrity of the tetrachlorobenzene ring. Notably, tetrachloronaphthalene was consistently absent in our analysis and in previous studies of PG7 fragmentation, whereas naphthalene and methylnaphthalene have always been detected.

### PCA, difference between pigment and ink

**Fig. 5:**
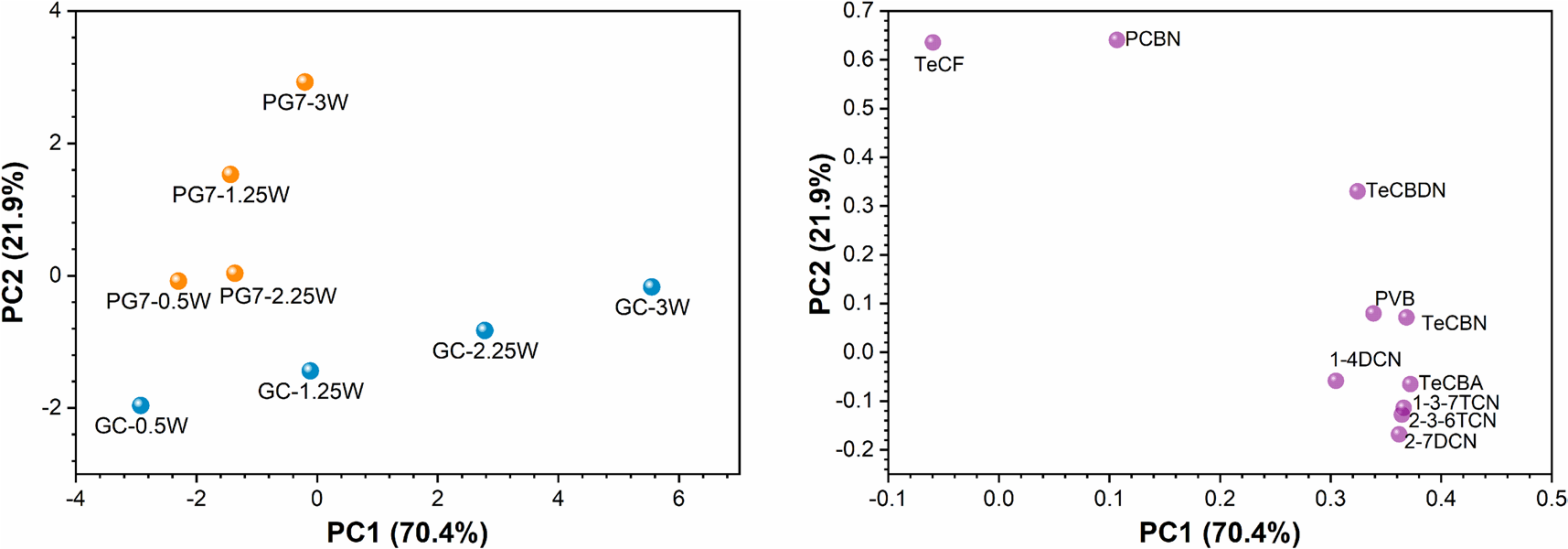
PCA of the GC and PG7 samples treated for 25 min: score plot (left panel) and loading plot (right panel), including the percentage of explained variance on each axis. The blue dots correspond to the GC, the orange dots correspond to PG7, and the violet dots correspond to the PCA weights of the fragments

Principal component analysis (PCA) was conducted to provide further information on the relationship between fragmentation and laser power for the GC ink and PG7 pigment samples. The analysis was conducted on the normalized peak areas of the fragments from both the GC ink and the PG7 pigment, which were treated for 25 min with laser powers ranging from 0.5 to 3 W (Fig. 5). Two fragments were excluded from the analysis: TCBN, due to its absence in the PG7 samples, and TeCHI, because it was present only in the G25-1.25 and G25-3 samples.

The analyses were conducted on normalized samples, which were centered and scaled to unit variance via the following equation:

$$Scaled X=\frac{{X}_{i} - mean\left(X\right)}{sd(X)}$$

where *X*_*i*_ represents the peak areas of the fragments, *mean(X)* represents the mean value averaged over the peak areas of *X* fragments and *sd(X*) represents the standard deviation of the normalized peak areas. This ensures that features with large variances do not dominate the PCA representation.

The score plot of the PCA revealed that the first principal component (PC1) accounts for 70.4% of the total variance between the samples, whereas the second principal component (PC2) explains 21.9%.

PC1 predominantly differentiates the GC samples on the basis of the applied power, with the samples arranged in order of increasing laser power along the horizontal PC1 axis. In contrast, the PG7 samples exhibit a weaker separation along this axis. However, the distinction becomes more pronounced for the PG7 samples along the second component (PC2), although it is not linear, as previously observed, but rather, the difference between the two extremes of applied power is considered.

The position of the majority of the fragments in the loading plot shows that, compared with that of the PG7 pigment, the fragment intensity increases with increasing power applied to the GC ink.

The separation along PC2 for the PG7 pigment samples is also explained by the positions of fragments TeCF and PCBN in the loading plot. Compared with those of the GC samples, the intensities of these peaks indicate greater fragmentation of PG7 at 3 W of power. Furthermore, the TeCBDN fragment in the loading plot indicates that its intensity increases with increasing applied power for both sample types.

PCA confirmed a stronger dependence of fragment intensity on power in the GC samples than in the PG7 samples, as an increase in power led to a corresponding increase in fragment production. Previous semiquantitative analyses revealed that, for six selected fragments, this increase followed a linear trend. The positioning of TeCBDN in the loading plot suggests that its production increases in response to increasing power across both sample types.

### SEM imaging

**Fig. 6:**
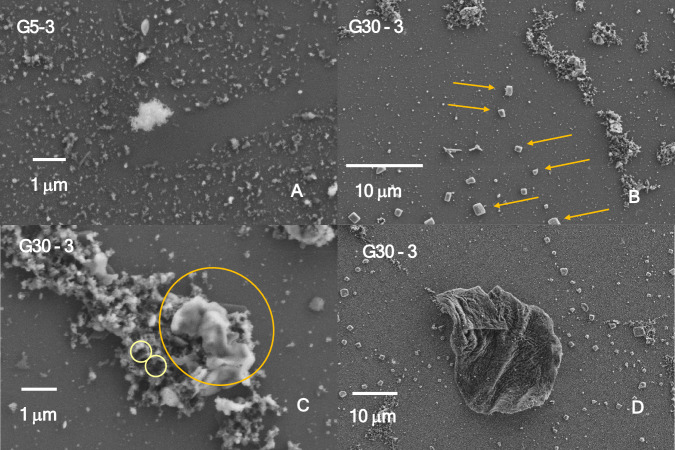

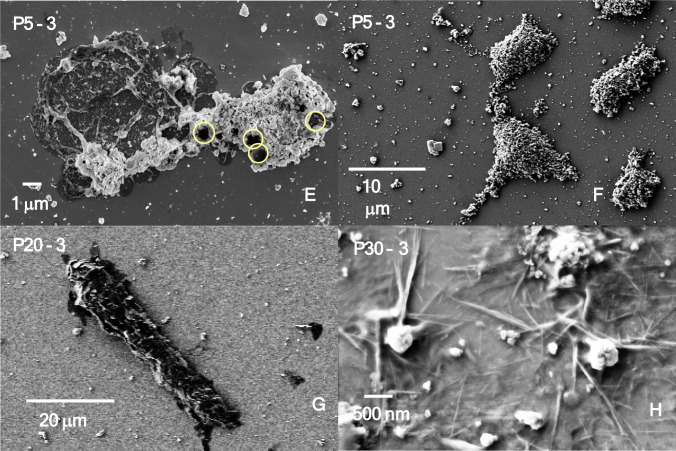
SEM images of representative samples treated with various powers and durations of irradiation with a Ti:sapphire femtosecond laser. GC ink was treated for **A** 5 min at 3 W and **B**, **C**, and **D** 30 min at 3 W (different areas). PG7 pigment treated for **E** and **F** 5 min at 3 W (different areas), **G** 20 min at 3 W, and **H** 30 min at 3 W

SEM images were collected from samples deposited on silicon wafers (used as sample holders) immediately at the end of each treatment, and as such, snapshots of the particle sizes and morphologies related to the processes triggered by exposure to the femtosecond laser were obtained. Figure 6 shows images of the most common particles, aggregates, filaments and composite structures found in both the GC and PG7.

In general, femtosecond laser treatment causes processes of fragmentation and melting, similar, to some extent, to those of nano- and picosecond lasers. However, peculiarities can be observed, both in terms of the shape and size distribution of the treated samples although distinctions must be made between GC and PG7. The most striking feature of all the GC-treated samples was the dominant presence of particles or small aggregates in the range between 20 and 120 nm, an example of which is reported in Fig. 6A. Their regularity is quite distinctive, since it could not be observed, to this extent, on equivalent samples treated with nano- and picosecond lasers (Cecchetti et al. 2022; Bauer et al. 2020). Simultaneously, larger particles form as a result of a melting process, as shown in Fig. 6B, C, highlighted by orange arrows or a circle. The latter, in particular, indicates a melted area emerging from the initial sheath of particles embedded in the ink, which forms islands. Larger agglomerates, with a diameter on the order of tens of microns, also form, as shown in Fig. 6D, and are more abundant with increasing power.

Finally, piercing of the initial sheath by the laser beam is observed at several points, two of which are marked by yellow rings in Fig. 6C.

**Fig. 7:**
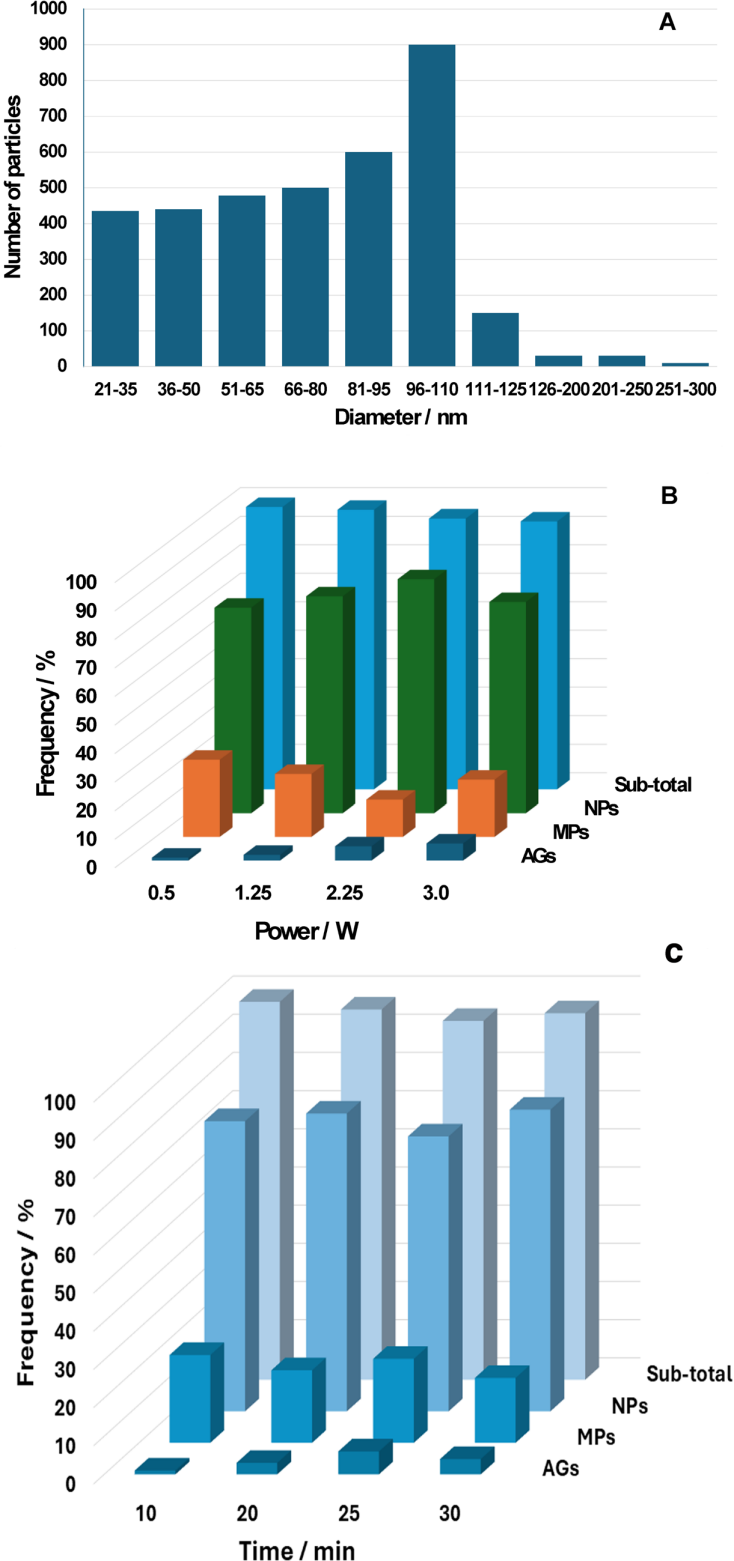
Quantitative assessment of the particle size distribution via SEM images of the treated samples. **A** Particle size distributions of the GC samples treated for 30 min at 2.25 W. 3D plot of the particle size distributions of the GC samples: **B** treated for 25 min with increasing laser power and **C** treated at 3 W with increasing time. The particles are categorized on the basis of their diameter into NPs (< 200 nm), MPs (201–500 nm), AGs (> 501 nm), and subtotal particles (sum of NPs and MPs)

A quantitative assessment of the particle size distribution was performed through statistical analysis of areas of 1 mm^2^ from SEM images of each sample, and the results are reported in Fig. 7A–C. Figure 7A presents an example of the particle size distribution in sample G30-2.25, which is in the 20–300 nm range and indicates an increasing frequency with diameter, with a peak in the 96–110 nm range, followed by a frequency drop, for particles larger than 110 nm. Notably, nanoparticles embedded in the sheath were excluded from the statistics, and for asymmetric particles (i.e., those with an aspect ratio > 1), the largest dimension was selected for counting. In addition, nanoparticles in the 2–3 nm range are also observed, but in small amounts, they are on the order of 1 every 50 with sizes > 20 nm. They have also been excluded from general counting because their identification requires higher magnifications, which results in the loss of an overview and the absence of correct counting. To assess the overall size distribution as a function of power and time, 3D plots were generated, where the particles were grouped into three categories, i.e., with diameters < 200 nm, 201–500 nm and > 501 nm and labeled NPs, MPs and AGs, respectively. The plot of the size distributions at 25 min and increasing power is shown in Fig. 7B, whereas the histogram at 3 W and increasing time is shown in Fig. 7C. In these analyses, the points after 5 min of treatment were excluded because of the prevalence of untreated sheaths in the images. The subtotal is the sum of the NP and MP frequencies. The trend observed as a function of power indicated a frequency peak of the NPs at 2.25 W and, correspondingly, a minimum of MPs at the same power. On the other hand, the trend of the subtotal is a monotonic decrease to a plateau that parallels a monotonic increase in the AGs. This is in line with the decrease in the absorbance of the UV‒Vis spectra with increasing power to a plateau, suggesting that the generation of particles < 500 nm is responsible for the spectral variations. The trend of the frequencies at 3 W with time has up-and-down trends for all categories. However, in general, an increase in the frequency of the NPs corresponds to a decrease in the number of MPs, and in this case, the subtotal frequency parallels the intensity of the spectral variations in the corresponding UV‒Vis spectra.

The SEM images obtained upon treatment of PG7 with the femtosecond laser reveal some peculiarities, largely related to the level of aggregation, which is much stronger than that of GC because of the inherent hydrophobicity of the pigment and its additives. This results in extended agglomerates, as shown in Fig. 6E, where beam piercing is also visible (marked by yellow rings). Islands also form in this case although they are larger than those in the GC. For comparison purposes, the average dimensions of the islands were estimated to be 2.5 ± 1.7 mm × 3.2 ± 2.1 mm for GC and 3.7 ± 2.0 mm × 8.3 ± 3.0 mm for PG7.

Agglomerates with a large aspect ratio (rods) form at the top power and intermediate exposure time (20 min, Fig. 6G, P20-3) and are similar to those observed by Kihara et al. (Kihara et al. 2019), who purposely grew long agglomerates upon nanosecond treatment followed by aging.

Most notably, thin fibers formed upon treatment of the PG7 at the top power (Fig. 6H, P30-3) and were present at all the different exposure times (whereas at the same level of treatment, the GCs displayed large agglomerates). Notably, a similar behavior, i.e., the appearance of fibers, was observed both upon Nd:YAG nanosecond laser treatment (3.5 J/cm^2^, 15 and 44 min) of the pigment PG36 (Bauer et al. 2021), which was dispersed either in water or in propan-2-ol, and from the extract of the GC, which was dispersed in water and subsequently treated with a Nd:YAG nanosecond laser (0.525 J/cm^2^, 120 min) (Bauer et al. 2020).

Nanoparticles ranging from 20 to 120 nm in size, as well as those on the order of 2–3 nm, are also observed, but with a far lower frequency than that of the GC. Owing to the large inhomogeneity of the formed particles as well as the large degree of agglomeration, a statistical analysis of the particles was not performed.

## Discussion

The comparative analysis of PG7 and GC upon femtosecond laser treatment revealed distinct behavior with respect to discoloration, the fragment production ratio, and the shape and size distribution of the generated particles. This is remarkable since GC is an ink containing PG7 as its sole pigment, and all differences are consequently related to additives.

First, discoloration is more efficient with GC than with PG7. The spectral UV‒vis intensities display an up-and-down variation with the treatment duration, which indicates the interplay of several factors, among which the downsizing of agglomerates with the intervention of the solvent plays a major role. Phthalocyanines are insoluble in nearly all solvents, and they can be dispersed to different degrees but are always small particles that are suspended in the liquid media. The intensity of the UV‒Vis spectra, therefore, comes from molecules on the surface of the particles: the larger the surface/volume ratio is, the more intense the spectrum. As a consequence, the size reduction caused by laser treatment actually increases the intensity of the spectra, as observed for many phthalocyanines, (Sugiyama et al. 2004; Omura et al. 2019; Rakov et al. 2020) the size of the particles being solvent dependent (Tamaki et al. 2003). In fact, spectral intensity augmentation is also used as a probe of agglomeration formation (Tamaki et al. 2002). Moreover, further morphological changes may take place following aging of the nanosecond laser-treated dispersions, as is the case for F_16_CuPc (hexadecafluoro copper phthalocyanine) (Kihara et al. 2019). In their investigation, Kihara et al. observed initial downsizing of microcrystals into nanometric spheres, followed by their rearrangement into rods upon aging, characterized by a further increase in spectral intensity. The growth was both temperature- and solvent dependent.

For the PG7 and GC samples treated by femtosecond lasers, the shape is highly dependent on the type of sample. SEM imaging reveals a prevalence of downsizing to spherical nanoparticles in the case of GC. Rods or needles are observed nearly exclusively in the PG7-treated dispersions. This can definitively reflect the dispersion of the particles generated upon treatment in a water solution, with consequent variable absorption of the UV‒vis spectra.

Toxic chlorinated fragments are generated by femtosecond laser treatment of CG and PG7, both derived from the phthalocyanine-based pigment and from naphthalene impurities present in both the pigment and the ink. This is similar to what was previously reported for nanosecond and picosecond laser treatments, thus implying that femtosecond laser treatments are not safer in this regard.

The toxicological aspects of all fragments directly related to the phthalocyanine macrocycle, such as chlorinated benzonitriles, benzodinitriles or phthalimides, have been discussed in our previous works (Bauer et al. 2020, 2021; Cecchetti et al. 2022). The compounds are mostly classified as skin, eye or respiratory irritants, but fragments such as trichlorobenzonitrile are toxic if swallowed or in contact with the skin. Prolonged or repeated exposure to chlorinated naphthalene may damage organs (H373) in addition to causing severe irritation (H373). Some major concerns are related to the treatment-dependent increase in severe irritants, i.e., TeCBN and TeCBDN, or compounds for which toxicological data are not available (TeCHI).

The yields of fragments and their relative ratios are also dependent on the sample, as well as on the irradiation conditions. In particular, the fragment yield varies significantly with laser power and shows a linear dependence on power, although only for GC and not for PG7. Furthermore, the percentage increase with increasing power also varies with the number of fragments, indicating activation through nonlinear optical processes. The treatments at fixed power and as a function of time produce fragments with oscillating yields. The PCA results indicate a stronger dependence of fragment intensity on power in the GC samples than in the PG7 samples, as an increase in power leads to a corresponding increase in fragment production.

To some extent, the variable yield of the phthalocyanine decomposition fragments can also be related to the morphology of the particles produced after the treatments, particularly the outcome of the entrapment of the generated fragments into the large agglomerates, which may hinder their detection. This, therefore, applies more strongly to PG7, which displays larger agglomerates than does GC, thus confirming the observed trend.

The substantially different behavior of PG7 and GC with respect to the femtosecond treatments opens up a completely new scenario regarding the safety of the removal process, as the outcome—in terms of the relative abundance of toxic fragments and the aspect ratio of the agglomerates—depends on the power and duration of the treatments, which is likely to also occur when applied to the skin. Furthermore, it may depend on the actual chemical and physical properties of the ink, i.e., whether the hydrophilic components have been stripped, causing it to behave more like PG7, or if it remains similar to the injected GC. As a consequence, its behavior may vary, depending on where it resides in the skin, i.e., in a more or less lipophilic region. In fact, although the ink, not the pigment alone, is injected under the skin, the actual agglomeration and effective hydrophobicity of the ink depend on the identity of the ink dispersants and soluble components due to skin metabolism resulting from the injection and possible compound release or retention (Benedetti et al. 2009). Furthermore, this implies that different outcomes may be related to different settings in the removal procedure when the laser beam is applied to the skin.

Finally, since 2022, PG7, along with all phthalocyanines, has been banned under the REACH regulations (Registration, Evaluation, Authorization, and Restriction of Chemicals) for use as a pigment in tattoo inks. However, removal issues are related to existing tattoos; hence, these methods are quite likely to be performed with by-now-banned pigments.

## Conclusions

The efficacy and potential harm of femtosecond laser treatments on green tattoo ink (GC) and its pigment PG7 were investigated, and the results highlight their distinct behaviors in terms of discoloration, toxic fragment production ratios, and the shape of the resulting particles.

UV‒vis analysis of the GC ink and PG7 pigment samples treated with femtosecond lasers revealed distinct patterns in spectral intensity based on the treatment conditions. For the GC ink, the spectral intensity decreases linearly with increasing laser power when the exposure duration is fixed at 25 min, with a similar but less-pronounced trend for the PG7 pigment. However, when the laser power is held constant at 3 W, the intensity fluctuates as the exposure time increases.

For GC, in general, the efficacy of discoloration can be compared to that of a nanosecond laser, taking into account the different irradiated volumes of solution.

A subsequent GC‒MS analysis identified two primary fragment types, all potentially harmful: those resulting from chlorinated phthalocyanine decomposition and those related to active chlorination of naphthalene impurities.

The presence of these moieties rules out a lesser harm of the femtosecond laser treatment, on the basis of processes triggered by the high laser power although the role of using an IR wavelength of the source must be taken into account.

Semiquantitative assessments were conducted by plotting normalized peak areas for selected fragments in response to varying irradiation powers (at a fixed 25-min exposure) and exposure durations (at a fixed 3 W power). For the GC ink, a clear linear increase in fragment intensity with increasing power level was observed, as shown by the high R^2^ values for the six key fragments, indicating a strong correlation between the applied power and the fragmentation rate. This linear trend was not consistently observed in the PG7 samples, nor was a linear relationship noted between the fragment intensity and increased exposure time, which aligns with the UV‒vis findings.

Production rates were calculated for fragments in GC ink, which clearly increased in a power-dependent manner. PCA further confirmed the strong dependence of fragment intensity on applied power in the GC samples compared with that in the PG7 samples. The positioning of TeCBDN in the loading plot suggests a consistent increase in its production with increasing power across both the GC and PG7 samples, indicating a power-responsive fragmentation pattern.

Nevertheless, the yield ratios of the various fragments differ between PG7 and GC, and as a result, the associated toxicity also varies.

The morphology of the generated particles differed significantly between the GC and PG7 samples. The irradiation of a GC with a femtosecond laser yields primarily nanoparticles with a rather homogeneous size distribution, which are typically considered nontoxic. Large aggregates are also formed but with a regular shape. PG7, instead, yields rods and needles with an aspect ratio that is similar to that of toxic fibers.

In perspective, further investigations are envisaged to elucidate the role of additives that cause differences between irradiation of the pigment and that of the ink that contains the same pigment.

## Supplementary Information

Below is the link to the electronic supplementary material.

Supplementary file1 (DOCX 1320 KB)

## Data Availability

The datasets generated and analysed during the current study are available from the corresponding author on reasonable request.
