# Supplementary material for: Toxicological problems of tattoo removal: characterization of femtosecond laser-induced fragments of Pigment Green 7 and Green Concentrate tattoo ink
Source: Arch Toxicol. 2025 Jan 15;99(4):1355–69. doi: 10.1007/s00204-024-03953-6 (PMC11968555; doi:10.1007/s00204-024-03953-6)
Supplement: Supplementary file 1 — Supplementary file1 (DOCX 1320 KB) [file 204_2024_3953_MOESM1_ESM.docx]

**Supplementary Information**

**Toxicological problems of tattoo removal: characterization of femtosecond laser-induced fragments of Pigment Green 7 and Green Concentrate Tattoo Ink**

Elvira Maria Bauer^1#^, Cosimo Ricci^2#^, Daniele Cecchetti^2#^, Giorgia Ciufolini^2^, Daniel Oscar Cicero^2^, Marco Rossi^3^, Ettore Guerriero^4^, Stefano Orlando^5^, Marilena Carbone^2*^

^1^ Institute of Structure of Matter - Italian National Research Council (ISM-CNR), c/o Area della Ricerca di Roma1, Strada Provinciale 35d n. 9, Montelibretti, Rome, 00010, Italy

^2^ *STARTNETICS* – Department of Chemical Science and Technologies, University of Rome Tor Vergata, Via della Ricerca Scientifica 1, 00133, Rome, Italy

^3^ Department of Basic and Applied Sciences for Engineering (SBAI), Sapienza University of Rome, Via Antonio Scarpa 16, 00161 Rome, Italy & Research Center on Nanotechnologies Applied to Engineering (CNIS), Sapienza University of Rome, P.le Aldo Moro 5, 00185 Rome, Italy

^4^ Institute for Atmospheric Pollution Research, Italian National Research Council (CNR-IIA), c/o Area della Ricerca di Roma1, Strada Provinciale 35d n. 9, Montelibretti, Rome, 00010, Italy

^5^ Institute of Structure of Matter - Italian National Research Council (ISM-CNR), FemtoLAB, C.da S. Loja, 85050 Tito Scalo, Italy

^#^ these authors equally contributed to the paper

^*^ Corresponding author carbone@uniroma2.it

Visual comparison of water dispersions of Green Concentrate treated with Ti:Sapphire femtosecond laser, Nd:YAG picosecond laser and Ruby nanosecond laser.

**Figure SI1**. Vials of GC dispersions upon different laser treatments: a) while undergoing Ti:Sapphire femtosecond lasing, b) upon Nd:YAG picosecond laser treatment, c) upon Ruby nanosecond laser treatment. The initial concentration of GC is the same for all three samples, as well as the total irradiation energy, though in a) the procedure is not completed yet.
